# Rabies in Pakistan: Policies and recommendations

**DOI:** 10.1002/puh2.168

**Published:** 2024-03-22

**Authors:** Abdul Jabbar, Attaullah Ahmadi, Nimra Irm, Iqra Naseeb, Shekiba Madadi, Don Eliseo Lucero‐Prisno

**Affiliations:** ^1^ Department of Veterinary Medicine Faculty of Veterinary Science University of Veterinary and Animal Sciences Lahore Punjab Pakistan; ^2^ Medical Research Center Kateb University Kabul Afghanistan; ^3^ Department of Quantitative Methods in Public Health École des Hautes Études en Santé Publique (EHESP) Paris France; ^4^ Department of Animal Breeding and Genetics University of Veterinary and Animal Sciences, Ravi Campus Pattoki Punjab Pakistan; ^5^ Institute of Microbiology Faculty of Veterinary Science University of Veterinary and Animal Sciences Lahore Punjab Pakistan; ^6^ Premier Cardiac and Vascular Fredericksburg Virginia USA; ^7^ Department of Global Health and Development London School of Hygiene and Tropical Medicine London UK

**Keywords:** dog bites, health policy, health system, Lyssavirus, Pakistan, rabies, zoonotic disease

## Abstract

Rabies, commonly referred to as the endemic disease of the impoverished in Pakistan, remains a significant public health concern. Globally, it contributes to over 55,000 fatalities annually, with 31,000 cases reported in Asia, predominantly among children. In Karachi, the yearly occurrence of rabies ranges between 7 and 9.8 cases per million individuals. However, the actual burden is likely underestimated due to underreporting. Key challenges in Pakistan include limited awareness and increasing dog bite incidents. To address this, comprehensive educational programs focusing on proper wound care, the ineffectiveness of homemade remedies, the importance of early medical intervention, and the administration of vaccines and post‐exposure prophylaxis are imperative. Implementing a One Health model, encompassing mass vaccination, cost‐effective treatments, training on intradermal vaccine administration, accurate data collection, and community awareness initiatives, is essential for rabies control. This article aims to provide policymakers and scholars with valuable insights into the primary factors and challenges of controlling endemic human rabies in Pakistan and proposes effective strategies for its control, with the ultimate goal of achieving rabies‐free status by 2030.

## INTRODUCTION

Rabies remains a lethal viral zoonosis, posing significant risks to global health. Transmitted primarily through animal bites, especially from infected dogs, the rabies virus threatens both human and animal populations worldwide. Rabies is transmitted from infected animals, particularly dogs, but can also be passed on by bats, foxes, raccoons, and other mammals to humans through bites [1]. Rabies accounts for over 55,000 fatalities annually worldwide, with 31,000 cases reported in Asia, primarily affecting children. Although developed countries with well‐established vaccination and animal control programs have witnessed a decline in rabies incidence, resource‐limited countries such as Pakistan continue to grapple with its menace, particularly affecting vulnerable populations with limited access to medical care and preventive measures. The “Zero by 30” global commitment launched in 2015 aims to eliminate rabies, emphasizing improved diagnosis, mass dog vaccination, and responsible pet ownership [2]. In this article, we provide an overview of the burden of rabies in Pakistan, its control measures and challenges against them, and recommendations to mitigate the burden of the disease in the country.

## RABIES EPIDEMIOLOGY IN PAKISTAN

Dogs predominantly account for 99% of the reported cases of rabies transmission to humans in Pakistan. In Karachi, the annual incidence is between 7 and 9.8 cases per million people [3]. Several hospitals in Rawalpindi city, situated in the Punjab province, report around 100 dog bite cases each month [4], with some government healthcare facilities attending an average of 50–70 individuals bitten by canines daily [5]. Annually, over 1.5 million cases of dog bites are documented, with a significant proportion occurring among children [6].

Alarmingly, 78% of the population in Pakistan lacks awareness regarding the fatality of rabies and the necessity of post‐exposure prophylaxis (PEP). Additionally, 42.7% of individuals are uninformed about the signs, symptoms, and treatments associated with this disease. Moreover, approximately 38.7% of the population rely on herbal remedies for treatment. The community's adherence to PEP with anti‐rabies vaccines is also suboptimal [7].

## RABIES CONTROL EFFORTS, CHALLENGES, AND RECOMMENDATIONS

The issue of rabies in Pakistan was first brought to attention during the First National Symposium on Rabies held at the National Health Laboratories in Islamabad in October 1969. Subsequently, an informal consortium known as the Asian Rabies Expert Bureau was formed to address rabies awareness and prevention among member countries. The “Rabies Free Karachi” campaign, launched in 2017, succeeded in the vaccination of 70% of dogs through WHO‐recommended mass dog vaccination efforts in Ibrahim Hyderi town [8]. This initiative also included strategies for animal birth control. In efforts to enhance human resources for rabies control, a pilot project was established in collaboration with the Indus Hospital Karachi and the University of Veterinary and Animal Health Sciences, Lahore [9].

Despite efforts taken to contain the disease, rabies continues to pose a public health threat in the country. Thus, concerted efforts and more initiatives are required to reach a rabies‐free Pakistan by 2030. Implementing large‐scale Animal Birth Control (ABC) programs, involving the capture, sterilization, vaccination, and release of stray dogs, is crucial [10]. Promoting responsible dog ownership is of paramount importance. Instead of resorting to inhumane methods of dog population control, emphasis should be placed on data collection, surveillance, and the vaccination and registration of animals [10]. Given that dogs are often found in areas abundant with food waste, managing household waste can help mitigate the population of free‐roaming dogs. Dog bite awareness campaigns in educational institutions, particularly schools, can significantly reduce incidents among young people. Additionally, recognizing that rabies also affects animals used for consumption or milk production, community awareness programs play a pivotal role in preventing human rabies, especially during disease outbreaks [11].The true extent of the rabies burden remains underestimated due to a substantial number of unreported cases. Surveillance data and statistics on dog bite injuries are essential for effective monitoring and epidemiological assessment [2]. Implementing population control measures for dogs should prioritize mass dog vaccination alongside targeted vaccination campaigns [8]. It is imperative that every district hospital has access to cost‐effective anti‐rabies vaccines and facilities for managing animal bite wounds. PEP should be readily available to high‐risk populations, such as animal handlers, rural residents engaged in outdoor activities, and laboratory staff. Addressing the lack of awareness necessitates comprehensive education programs on rabies. Communities should be discouraged or prohibited from using unproven remedies at home [12].The aforementioned recommendations are vital to be implemented as many government hospitals in Pakistan are underfinanced and thus unable to afford the treatment of dog bite victims [13]. Pakistan's healthcare system confronts significant challenges due to limited infrastructure and restricted access to government‐provided resources, particularly in rural areas. Shortages of rabies vaccines and rabies immunoglobulin (RIG) have been documented in various regions of the country. RIG vaccines are often prohibitively expensive and predominantly available in the private healthcare sector [1]. The government should promote local production of anti‐rabies vaccines to address issues related to vaccine importation from India and cater to local demand. Currently, most rabies centers utilize a five‐dose intramuscular regimen, but transitioning to the more cost‐effective intradermal four‐dose regimen could result in approximately 80% cost savings. This regimen is strongly recommended due to its cost‐effectiveness compared to other regimens. Last but not least, health personnel need proper training to effectively counsel patients on compliance with the PEP regimen [14].

## CONCLUSION

Rabies management remains a formidable public health challenge in Pakistan, necessitating a unified One Health approach and the effective implementation of national rabies control programs. Achieving zero deaths from rabies by 2030 requires coordinated and strategized efforts through inter‐regional and inter‐sectoral collaborations. Key interventions include dog population control measures, mass vaccination campaigns, and enhanced public awareness of PEP.

## AUTHOR CONTRIBUTIONS

Don Eliseo Lucero‐Prisno III, Abdul Jabbar, and Attaullah Ahmadi conceived the idea. Abdul Jabbar, Nimra Irm, Iqra Naseeb, Shekiba Madadi, and Attaullah Ahmadi drafted the manuscript. Don Eliseo Lucero‐Prisno III assisted in article interpretation and language editing. All the authors read and approved the final manuscript.

## CONFLICT OF INTEREST STATEMENT

Attaullah Ahmadi is a Youth Editorial Board member of Public Health Challenges and a co‐author of this article. Don Eliseo Lucero‐Prisno III is Editor‐in‐Chief of the journal and a co‐author of this article. They were excluded from the peer‐review process and all editorial decisions related to the acceptance and publication of this article.

## FUNDING INFORMATION

None.

## ETHICS STATEMENT

Not applicable.

## Data Availability

Not applicable—no new data generated.
